# Wireless wide-range pressure sensor based on graphene/PDMS sponge for tactile monitoring

**DOI:** 10.1038/s41598-019-40828-8

**Published:** 2019-03-08

**Authors:** Hairong Kou, Lei Zhang, Qiulin Tan, Guanyu Liu, Helei Dong, Wendong Zhang, Jijun Xiong

**Affiliations:** Science and Technology on Electronic Test and Measurement Laboratory, North University of China, Tai Yuan, 030051 China

## Abstract

We propose a flexible wireless pressure sensor, which uses a graphene/polydimethylsiloxane (GR/PDMS) sponge as the dielectric layer. The sponge is sandwiched between two surfaces of a folded flexible printed circuit with patterned Cu as the antenna and electrode. By adjusting graphene and NH_4_HCO_3_ concentrations, a composite with 20% concentration of NH_4_HCO_3_ and 2% concentration of graphene as the dielectric layer is obtained, which exhibits high sensitivity (2.2 MHz/kPa), wide operating range (0–500 kPa), rapid response time (~7 ms), low detection limit (5 Pa), and good stability, recoverability, and repeatability. In addition, the sensor is sensitive to finger bending and facial muscle movements for smile and frown, that are transmitted using wireless electromagnetic coupling; therefore, it has potential for a wide range of applications such as intelligent robots, bionic-electronic skin and wearable electronic devices.

## Introduction

Flexible pressure sensors have been widely used in applications such as electronic skin, intelligent robots, and wearable devices^1–7^. These sensors have high-sensitivity, strong-flexibility, and implantable and wearable characteristics. Previous studies have reported three major conversion mechanisms to transform external pressure signals into electrical signals, namely piezoresistivity^8–10^, capacitance^11–14^, and piezoelectricity^15,16^. In all cases, high-sensitivity, low detection limit, fast response time, and low cost are the most desirable characteristics of flexible pressure sensors.

Sensitivity is one of the most important indicators to measure the working efficiency and measurement accuracy of pressure sensors. Researchers use a variety of methods to increase sensor sensitivity, such as adopting micro-nano structures to increase the contact area, searching for new composites to increase the detection range, and exploring new working principles for the sensor. Adoption of micro-nano structures is the most practical way to improve the sensing performance. Micro-nano structures, such as pyramids^17,18^, leaf textures^19,20^, spongy structures^21–24^, silk^3,25^, hollow hemispheres^26^, micropillar arrays^27^, and rugged structures^28^, have been proposed to improve the sensitivity of pressure sensors. New high-sensitivity composite materials are widely used in pressure sensors, such as graphene^10,21,24,25^, metal nanowire^27,29^, carbon nanotubes^3,11,23^, and other novel materials^30,31^.

However, the above-mentioned sensors require wire connections for data transmission. Wireless and passive sensors will be the trend of the future due to their long-distance transmission and battery-free operation. Inductor-capacitor (LC) technology with its small size, low cost, high stability, and battery-free operation has become the best option for wireless transmission. Hitherto, it has been applied for wireless measurement of pressure^32–38^, temperature^39,40^, humidity^41^, and specific components in sweat^42^.

Herein, we propose a wireless flexible pressure sensor based on a graphene/PDMS (GR/PDMS) sponge as a dielectric layer, which is sandwiched between the folded surfaces of a flexible printed circuit with Cu pattern as the antenna and electrode. First, the high-performance GR/PDMS sponge with high sensitivity, large operating range, rapid response time, low detection limit, good stability, recoverability, and repeatability was fabricated by adjusting graphene and NH_4_HCO_3_ concentrations. Second, a wireless flexible pressure sensor was fabricated and its working principle was introduced through a series of formulas and equivalent circuit diagram, which clearly explain the wireless operation of the LC sensors. Finally, to confirm the practicality of the fabricated wireless pressure sensor, we measured the capacitance and frequency response curves of our sensor. The fabricated sensor has the potential to be used in highly sensitive wireless detection for a wide range of applications, such as intelligent robots, bionic-electronic skin, and wearable electronic devices.

## Results and Discussion

### GR/PDMS sponge preparation and characterization

**Figure 1:**
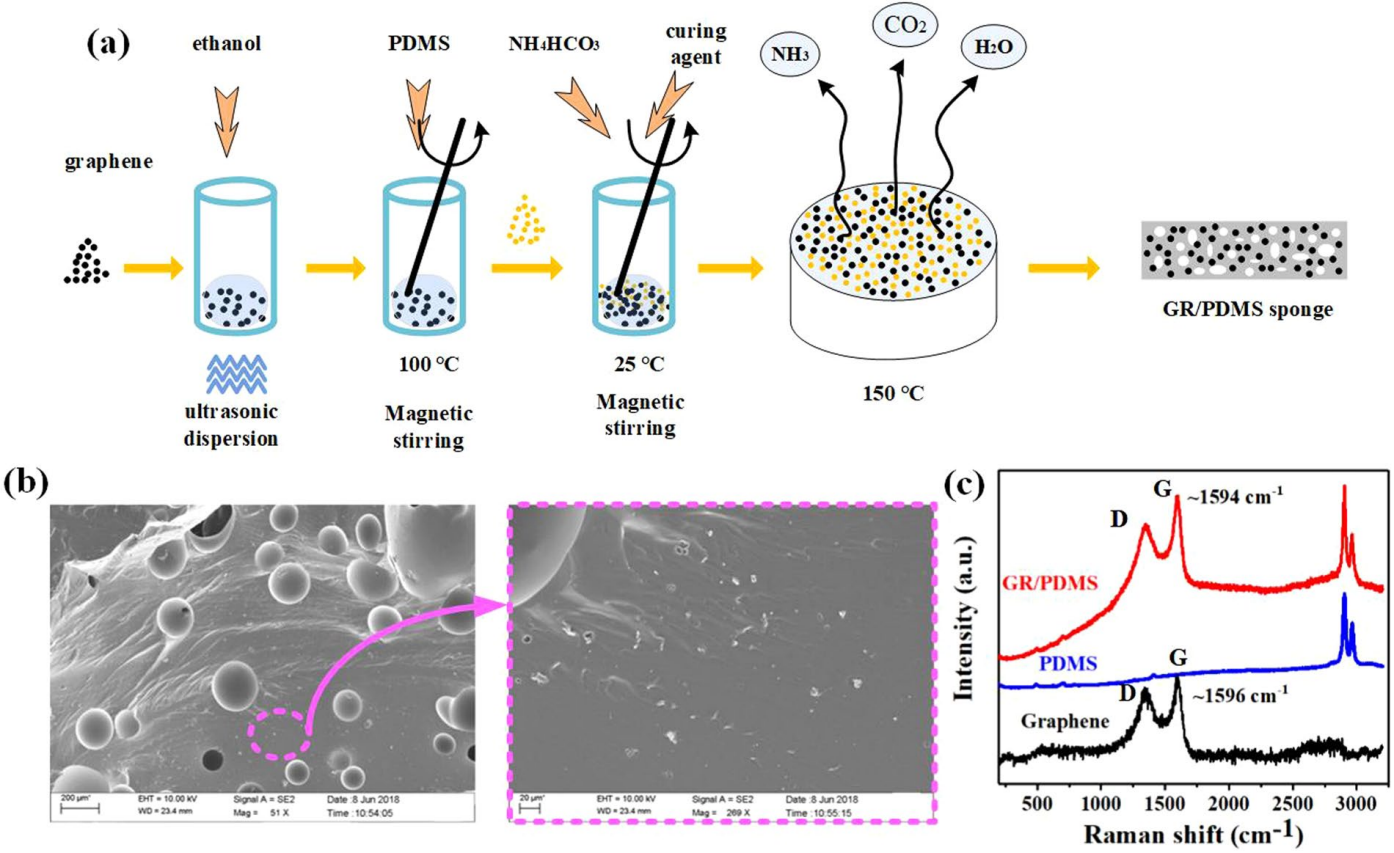
(**a**) Schematic illustration of the fabrication process of GR/PDMS sponge. (**b**) SEM image of GR/PDMS sponge. (**c**) Raman spectrum of graphene, PDMS, and GR/PDMS sponge.

The manufacturing process of the GR/PDMS sponge is shown in Fig. 1(a). The GR/PDMS sponge production steps are as follows: first, graphene was dispersed uniformly in ethanol by ultrasonic dispersion at 30 °C for 8 h to weaken the interaction between the graphene nanoparticles and obtain a good graphene suspension. Second, the PDMS main agent was added to the graphene suspension and heated at 100 °C until the ethanol was completely evaporated. After cooling, NH_4_HCO_3_ powder and PDMS curing agent were added to the previous mixture using magnetic stirring to uniformly disperse NH_4_HCO_3_ powder. Then, the mixture was poured on glass and heated at 150 °C until the NH_3_, CO_2_, and H_2_O were completely evaporated. According to Eq. (1) below, NH_4_HCO_3_ on heating will break down into NH_3_, CO_2_, and H_2_O. The density of the PDMS was simply modified by the gas evaporation, forming a GR/PDMS sponge of high deformation with air microfeatures. Using the above approach, dielectric layers with different concentrations of NH_4_HCO_3_ and graphene were fabricated. The fabricated GR/PDMS sponge can be cut into small pieces for use in the dielectric layer. The surface topography of the GR/PDMS sponge was obtained by SEM, and it was found that air holes were evenly spread across the PDMS; further, the graphene particles did not agglomerate and were uniformly distributed in PDMS, as shown in Fig. 1b.

1 $${{\rm{NH}}}_{{\rm{4}}}{{\rm{HCO}}}_{{\rm{3}}}\triangleq {{\rm{NH}}}_{{\rm{3}}}\uparrow +{{\rm{H}}}_{{\rm{2}}}{\rm{O}}\uparrow +{{\rm{CO}}}_{{\rm{2}}}\uparrow $$

The Raman spectrum of graphene, PDMS, and GR/PDMS sponge are shown in Fig. 1c. The Raman spectrum of the GR/PDMS sponge shows a D peak at 1344 cm^−1^, a G peak at 1594 cm^−1^, and two peaks within 2800 cm^−1^ to 3000 cm^−1^, which is the superposition of the Raman spectrum of graphene and PDMS.

### Sensor fabrication and working principle

**Figure 2:**
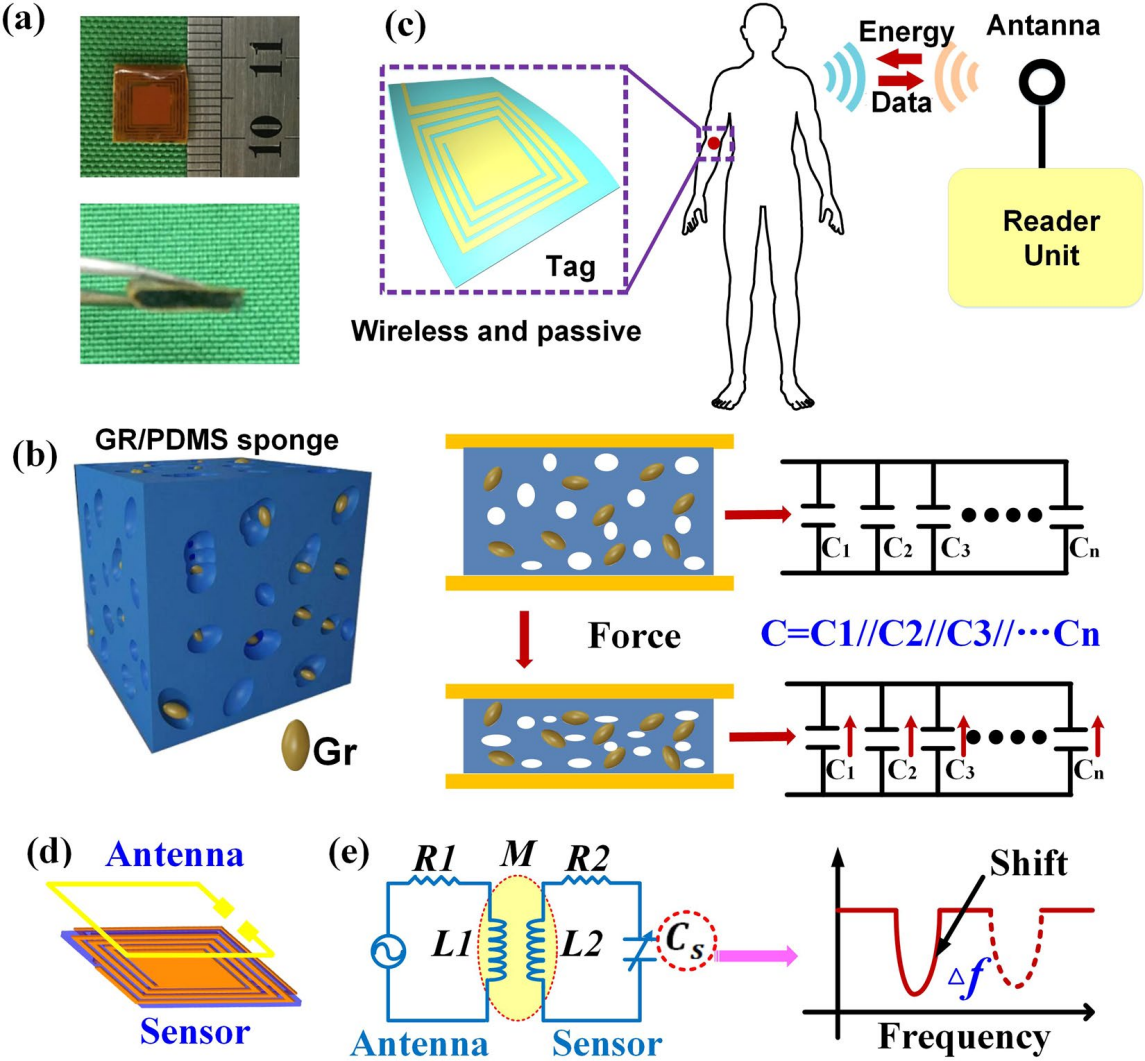
Wireless pressure sensor based on GR/PDMS sponge. (**a**) Photograph of wireless pressure sensor. (**b**) Schematic showing the configuration of the GR/PDMS sponge under force. (**c**) Schematic diagram of a wireless system. (**d**) Conceptual diagram of an LC wireless pressure sensor. (**e**) The equivalent circuit and resonant frequency variation of the wireless pressure sensor.

The flexible pressure sensor was fabricated by folding a flexible substrate with Cu circuitry and using the GR/PDMS sponge placed between the folded surfaces as the dielectric layer^43,44^, as shown in Fig. S1. The size of the packaged sensor is 9 × 9 × 2 mm, as shown in Fig. 2a. When external pressure is applied to the sensor, the GR/PDMS sponge is compressed, causing the air-holes to shrink. According to Eq. (2) ^45^, the increase in capacitance of the GR/PDMS sponge is mainly attributed to the decrease in the distance between the two electrode plates. To better explain the pressure sensing mechanism, we assume that both air-holes and graphene distributed in the PDMS play important roles when the sensor is under pressure. The graphene particles are separated by PDMS and the air-holes to form numerous parallel capacitors (C_1_,C_2_,C_3_•••C_n_), as depicted in Fig. 2b. The presence of the air-holes makes the sensor more susceptible to deformation when it is under pressure, resulting in a decrease in the space between the graphene particles. The total capacitance increases with the simultaneous increase in the number of parallel capacitors.

2 $${\rm{\Delta }}{\bf{C}}=\frac{{\boldsymbol{\varepsilon }}A}{{\rm{\Delta }}{\boldsymbol{d}}}=\frac{{{\boldsymbol{\varepsilon }}}_{0}{\rm{\Delta }}{{\boldsymbol{\varepsilon }}}_{{\boldsymbol{r}}}{\boldsymbol{A}}}{{\rm{\Delta }}{\boldsymbol{d}}}={\rm{\Delta }}{{\boldsymbol{C}}}_{1}+{\rm{\Delta }}{{\boldsymbol{C}}}_{2}={\boldsymbol{\delta }}{\bf{h}}\frac{\partial {\boldsymbol{C}}}{\partial {\boldsymbol{h}}}({\boldsymbol{h}},{\boldsymbol{\varepsilon }})+{\boldsymbol{\delta }}{\boldsymbol{\varepsilon }}\frac{\partial {\boldsymbol{C}}}{\partial {\boldsymbol{\varepsilon }}}({\boldsymbol{h}},{\boldsymbol{\varepsilon }})$$

The principle of the wireless system is shown in Fig. 2c. The LC tags based on the GR/PDMS sponge can be used for large and subtle human body motion detection, owing to the high sensitivity and wide sensing range. Figure 2d illustrates the working principle of the wireless pressure sensor. The antenna connected to the network analyser can transmit a series of sweep signals, containing the resonant frequency of the sensor. Thus, it can be seen from Fig. 2e, that the resonant frequency of the sensor decreases as the capacitance increases, which is in accordance with Eq. (3). The resonant frequencies of the sensor are wirelessly transmitted to the antenna through signal coupling. The resonant frequencies will be obtained by analysing the S11 parameters, and the relationship between the resonant frequency and the external pressure can be established.

3 $${\boldsymbol{f}}=\frac{1}{2{\boldsymbol{\pi }}\sqrt{{\boldsymbol{LC}}}}$$

### Characterization of GR/PDMS sponge

**Figure 3:**
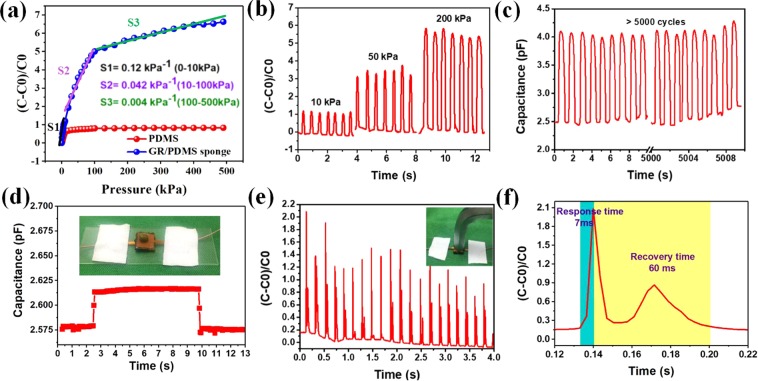
Capacitance variation characteristics of the GR/PDMS sponge under pressure. (**a**) Capacitance response curve with PDMS and GR/PDMS sponge as a dielectric layer under pressure of 0–500 kPa (**b**) Dynamic loading/unloading pressure with different load values. (**c**) Capacitance response curve of the pressure sensor over 5000 cycles. (**d**) Capacitance–time curve with loading and unloading of a mung bean. (**e**) Hitting the sensor with a hammer. (**f**) The response time and recovery time.

To characterize the performance of the GR/PDMS sponge, we measured the sensor sensitivity, response time, repeatability and detection limits using the platform we built with an impedance analyser (N4990A) and pressure gauge. The capacitance variation ratio ((C-C0)/C0, where C0 and C represent the initial capacitance and the capacitance under applied pressure) was plotted as a function of the applied pressure. The sensitivity is defined as S = δ(∆C/C0)/δP, which is a slope of the measured curve. When the NH_4_HCO_3_ concentration is 20% and graphene concentration is 2%, the sensor has the highest sensitivity, as shown in Fig. S2. The sensitivity curves with PDMS and GR/PDMS sponge as a dielectric layer are shown in Fig. 3a. The fabricated sensor could operate over a wide range of 0–500 kPa, which is a large operating range when compared with other reported sensors^18,19,24,27,33–35^. The sensitivity is 0.12 kPa^−1^ in the low-pressure range of 0–10 kPa, 0.042 kPa^−1^ for 10–100 kPa, and 0.004 kPa^−1^ in the high-pressure range of 100–500 kPa. The pressure sensor with GR/PDMS sponge as a dielectric layer shows an improved sensitivity compared with that using PDMS as a dielectric layer. In view of elastic nature of the as-prepared GR/PMDS sponge, according to the theory of mechanics of materials, the deformation of GR/PDMS at 0–100 kPa was proportionally conformed with the pressure applied on the capacitance plate which is known as Hooke Law. Therefore, the variation of capacitance in 0–100 kPa was much more significant and the pressure sensor shows a high sensitivity in 0–100 kPa, as shown in Fig. 3a. As the pressure exceeds 100 kPa, the as-prepared GR/PMDS sponge was in yield stage and at this stage the deformation of GR/PDMS sponge was low, which would then cause a low capacitance change and a low-level sensitivity.

In addition to the sensitivity, the stability, recoverability, and repeatability of a sensor are important parameters to characterise its performance. The sensor exhibits favourable recoverability when pressure is applied and released (Fig. S3). Figure 3b depicts the response curves with different pressure values (10, 50, and 200 kPa) when dynamic loading/unloading pressure is applied on the sensors. The fabricated sensor exhibits a consistent response value during the seven processes of loading/unloading under each pressure. This indicates that the sensor has excellent stability and repeatability. In order to further characterize the repeatability of the sensor, it was subjected to more than 5000 cycles of loading/unloading experiments under the same pressure, as shown in Fig. 3c, which confirm the high robust performance of this pressure sensor.

The parameters of the detection limit and response time were also analysed to further elucidate the performance of the fabricated sensor. A mung bean with a mass of 50 mg was used as the pressure source (~5 Pa) and placed on the surface of the pressure sensor. Figure 3d indicates that the sensor can detect a lightweight object and output a response signal because of the presence of air-holes. An endurance test was performed by hitting the sensor with a hammer. The sensor quickly restored to its initial state after each hit, as shown in Fig. 3e, which further explains that the sensor has good recovery and endurance. More information can be derived from Fig. 3e, as shown in Fig. 3f. The response time is 7 ms and the recovery time is 60 ms, which suggests an instantaneous response when compared with other reported sensors^8,13,24,27^. The sensor can sensitively detect the bending motion of a finger as shown in Fig. S4.

### Characterization of resonant frequency response

From the measured results mentioned above, the GR/PDMS sponge as a dielectric layer exhibits the excellent properties of a wide operating range, rapid response time, low detection limit, good stability, recoverability, and repeatability. For further wireless testing of the practicality of the sensor, we built a wireless test system, as shown in Fig. S5.

**Figure 4:**
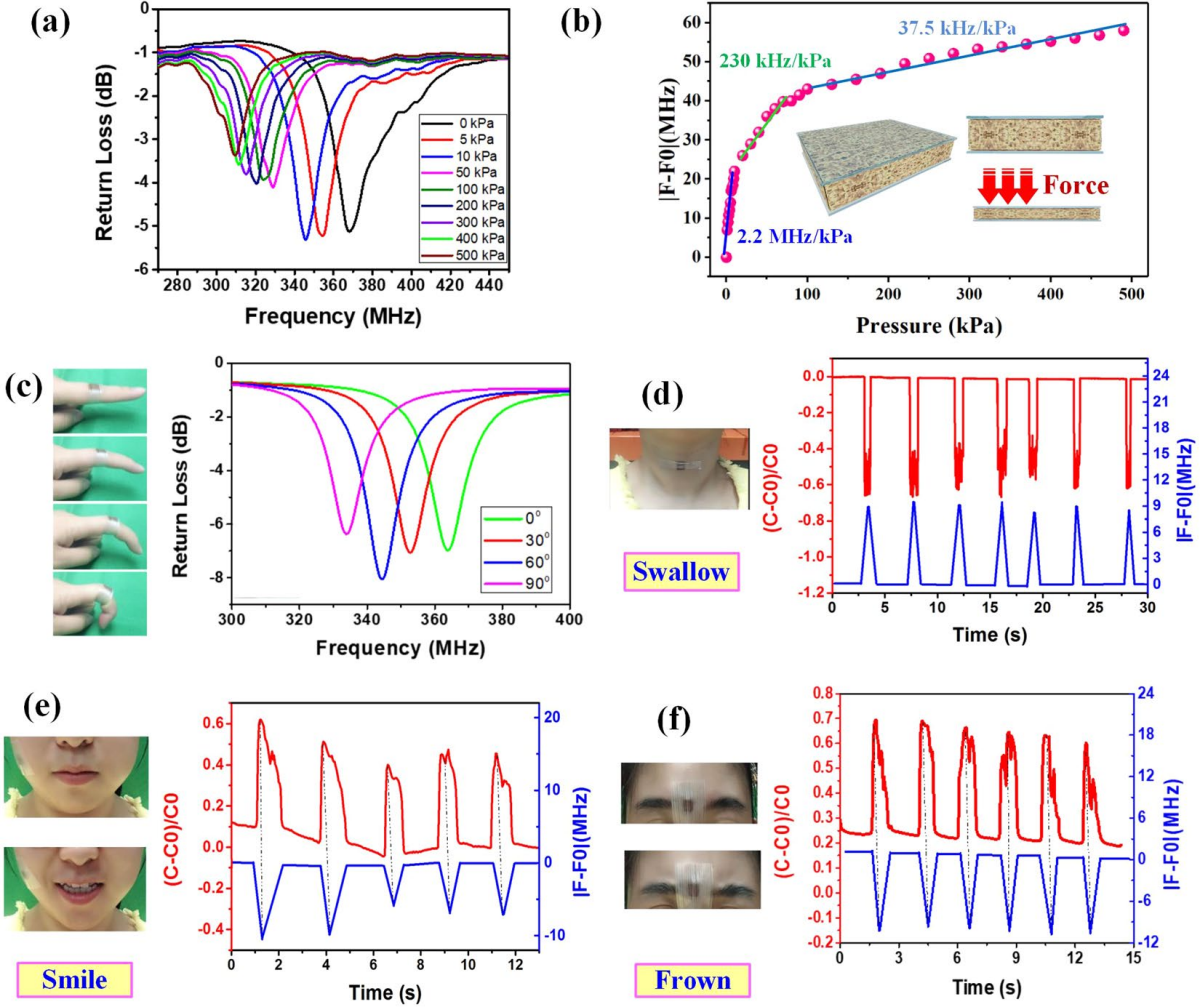
Frequency variation characteristics of the wireless pressure sensor. (**a**) Frequency curves under different pressures. (**b**) Measured frequency changes as a function of the applied pressure for the wireless pressure sensors (**c**) Resonant frequency curves corresponding to the state of the finger bending. Capacitance response curve and frequency response curve for (**d**) swallow, (**e**) smile, and (**f**) frown.

Figure 4a shows the corresponding output frequency curves under different external pressures. There is a unique minimum in the pressure range of 0–500 kPa for each curve, which corresponds to the resonant frequency of the sensor. The resonant frequency of the sensor is reduced from 367.67 to 309.19 MHz as the external pressure increases from 0 to 500 kPa. The absolute value of the difference between the initial and deformed resonant frequency points extracted from the curves with different external loading values were plotted against different pressure in Fig. 4b. The sensitivity is 2.2 MHz/kPa in the low-pressure regime (0–10 kPa), 230 kHz/kPa in the middle-pressure regime (10–100 kPa), and 37.5 kHz/kPa in the high-pressure regime (100–500 kPa). The variation trends of the resonant frequency and the capacitance are approximately the same under the same pressure, which establishes the correctness and feasibility of the wireless detection method, as shown in Fig. S6.

To verify the practicality of the fabricated wireless pressure sensor, it was attached onto a finger to measure the finger bending movements. Each finger movement state corresponds to a resonant curve. When the finger was bent from the horizontal state (0°) to the vertical state (90°) at intervals of approximately 30°, the resonant frequency was changed from 363.91 to 333.89 MHz, as shown in Fig. 4c. That is because the bending of the finger causes an increase in the capacitance of the GR/PDMS sponge, resulting in a decrease in the resonant frequency of the sensor.

To further investigate the benefits of our wireless pressure sensor, we attached our sensors to the throat, face, and centre of the forehead to monitor the swallowing action, facial muscle movements for smile and frown. The demonstrations involved a healthy female volunteer with approval from the institutional review board of the North University of China. In addition, we confirmed that all experiments were performed in accordance with the relevant guidelines and regulations of the institutional review board. First, the capacitance response curve of the pressure sensor was obtained for repeated swallowing actions. Meanwhile, the resonant frequency curve (Fig. S7) was recorded by extracting the smallest point in the curve during the swallowing process, as shown in Fig. 4d. It can be concluded that there is a corresponding relationship between the capacitance response curve and the frequency response curve from Eq. (3). That is, the capacitance variation can be transformed into frequency variation in the wireless method. The capacitance and frequency response curves of the smile and frown processes were recorded using the previous method, as shown in Fig. 4e, and f. Therefore, our wireless pressure sensor can wirelessly detect finger bending and facial muscle movements, compared to other existing cabled sensors^3,8–26^, which gives it the potential to be used for highly sensitive wireless detection in a wide range of applications such as intelligent robots, bionic-electronic skin and wearable electronic devices.

## Conclusions

In summary, we propose a wireless flexible pressure sensor based on a GR/PDMS sponge as a dielectric layer, which is sandwiched by folding the flexible printed circuit with patterned Cu as the antenna and electrode. The GR/PDMS sponge with NH_4_HCO_3_ concentration of 20% and graphene concentration of 2% as the dielectric layer exhibits high performance with high sensitivity, wide operating range (0–500 kPa), rapid response time (~7 ms), low detection limit (5 Pa), and good stability, recoverability, and repeatability. The sensitivity is 2.2 MHz/kPa in the low-pressure regime (0–10 kPa), 230 kHz/kPa in the middle-pressure regime (10–100 kPa), and 37.5 kHz/kPa in the high-pressure regime (100–500 kPa). In addition, the practicality of the fabricated wireless pressure sensor was confirmed by capacitance and frequency response curves mapping for finger bending and facial muscle movements for smile and frown. Moreover, the sensor has the advantages of low cost, simple testing, high stability, and battery-free, and is thus suitable for use in highly sensitive wireless detection devices in a wide range of applications such as intelligent robots, bionic-electronic skin and wearable electronic devices.

## Methods

The raw materials needed for preparing the GR/PDMS sponge are graphene powder (thickness = 0.55–3.74 nm, diameter = 0.5–3 μm, number of layers ≤ 10, purity ≥ 98%), colloidal PDMS polymers(Sylgard 184, Dow Corning, Midland, MI, USA) PDMS curing agent, and NH_4_HCO_3_ power (a food additive).

The topography of the GR/PDMS sponge was characterized by using a scanning electron microscope (SEM) (Hitachi S-4800). The Raman measurements were taken with InVia Raman Microscope. The Raman spectrum were obtained using a 5 mW, 514.5 nm laster with a 50X objective. The capacitance-characterizations of the GR/PDMS sponge were performed using an impedance analyser (Agilent E4990A.) The frequency-characterizations of the wireless pressure sensor were performed using a vector network analyser (Agilent E5061B).

All data generated or analysed during this study are included in this published article. In this study, the experiments involved a volunteer with approval from the institutional review board of the North University of China. Their rights were protected and all subjects provided written informed consent to participate. The specific consents from the authors and volunteer have been obtained to publish the information in an open-access online publication. The authors confirmed that all experiments were performed in accordance with the relevant guidelines and regulations of the institutional review board.

## Supplementary information

Related Manuscript File
