# Supplementary material for: Wireless wide-range pressure sensor based on graphene/PDMS sponge for tactile monitoring
Source: Sci Rep. 2019 Mar 8;9:3916. doi: 10.1038/s41598-019-40828-8 (PMC6408520; doi:10.1038/s41598-019-40828-8)
Supplement: Supplementary file 1 — Related Manuscript File [file 41598_2019_40828_MOESM1_ESM.docx]

Electronic Supporting Information

**Wireless wide-range pressure sensor based on graphene/PDMS sponge for tactile monitoring**

Hairong Kou, Lei Zhang, Qiulin Tan*, Guanyu Liu, Helei Dong, Wendong Zhang, Jijun Xiong

Science and Technology on Electronic Test and Measurement Laboratory, North University of China, Tai Yuan 030051, China;

* Correspondence: tanqiulin@nuc.edu.cn;

**Wireless flexible pressure sensor fabrication**

The wireless pressure sensor consists of GR/PDMS sponge and flexible PI substrate with Cu electrodes. We propose a flexible high-performance pressure sensor using GR/PDMS sponge as the dielectric layer, which is sandwiched by folding the PI substrate with patterned Cu as the antenna and electrode, as shown in Fig. S1. This production process has the advantages of low cost, small size and easy fabrication.

Figure S1. The fabrication of wireless flexible pressure sensor.

**Characterization of GR/PDMS sponge**

Before the PDMS was dropped with graphene, four composite films with different concentration NH_4_HCO_3_ were fabricated. Fig. S2a illustrates that the sensor with 20% concentration NH_4_HCO_3_ has higher sensitivity because of the overall gas volatilization when the NH_4_HCO_3_ concentration is 40%. Then, by adjusting the graphene concentrations while maintaining the concentration of NH_4_HCO_3_ at 20%, GR/PDMS sponges with different graphene concentrations of 1%, 2%, and 4% were obtained. Fig. S2b shows the capacitance variation ratios of various GR/PDMS sponges with different applied pressure. A high performance sensor was achieved with a graphene concentration of 2%. When the graphene concentration is increased to 4%, the graphene particles, which are uniformly distributed across the PDMS, crosslink and undermine the capacitance transmission principle under the applied pressure, resulting in a poorer sensitivity, as reported in our previous work.^1^

Figure. S2. (a) Capacitance changes with the load pressure under the different NH_4_HCO_3_ concentrations. (b) Capacitance changes with the load pressure under the different graphene concentrations.

Fig. S3 shows the capacitance variation curves as a function of pressure under the process of applying pressure and releasing pressure. The sensor exhibits the excellent stability and recoverability.

Figure. S3. Applying pressure and releasing pressure.

To confirm the benefits of our pressure sensor with GR/PDMS sponge, the sensor was fixed onto a finger to measure the finger bending movements. When the finger was changed to bending and stretching, the variation of the pressure resulted in a variation of capacitance. A capacitance variation curve was plotted when the finger was bent from horizontal state (0˚) to the vertical state (90˚) in intervals of about 30°, as shown in Fig. S4. The pressure can detect the finger bending sensitively.

Figure. S4. Application of the proposed sensor in the detection of finger bending.

**Characterization of Resonant Frequency Response**

The test system comprised four parts: pressure sensor, interrogation antenna, network analyzer, and pressure gauge, The pressure sensor was provided energy from the interrogation antenna connected to network analyzer using the electromagnetic induction. A scanned alternate signal is input to the interrogation antenna, and the electromagnetic coupling of the wireless transmission is coupled to the internal circuit of the sensor. The pressure sensor resonates when the frequency of the sweep signal is the same as the self-resonant frequency of the pressure sensor. When external pressure is applied, the capacitance changes; causing the resonance frequency of the sensor to shift.

Figure. S5. Photograph of the measurement setup.

The capacitance and resonant-frequency response as a function of pressure were plotted, as shown in Fig. S6.

Figure. S6. The variation of resonant frequency and capacitance of the sensor as a function of pressure.


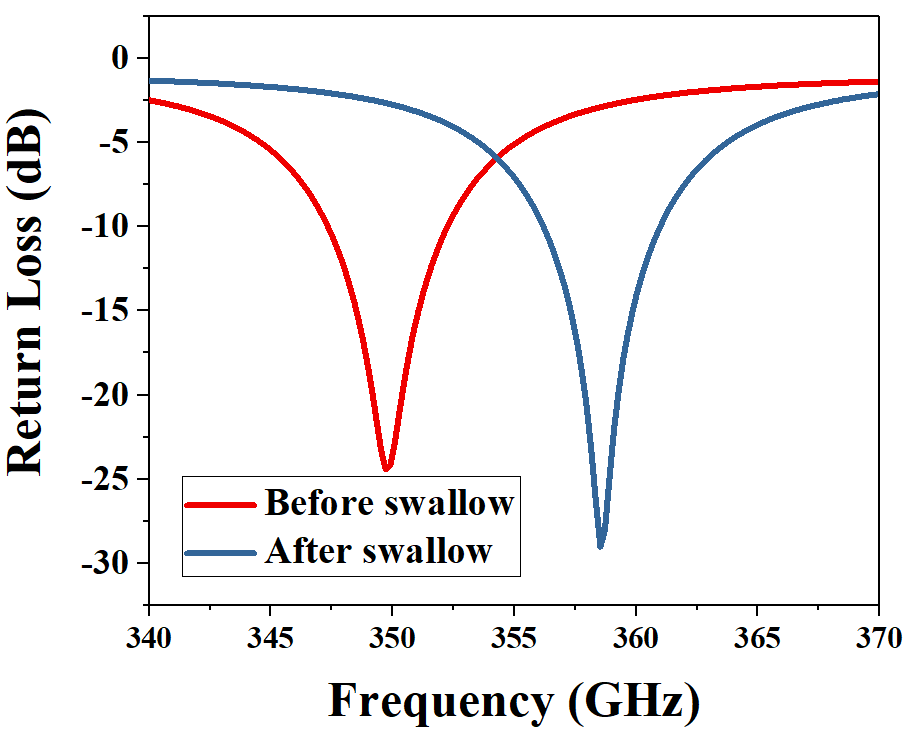


Figure. S7. The variation of resonant frequency before and after swallow.


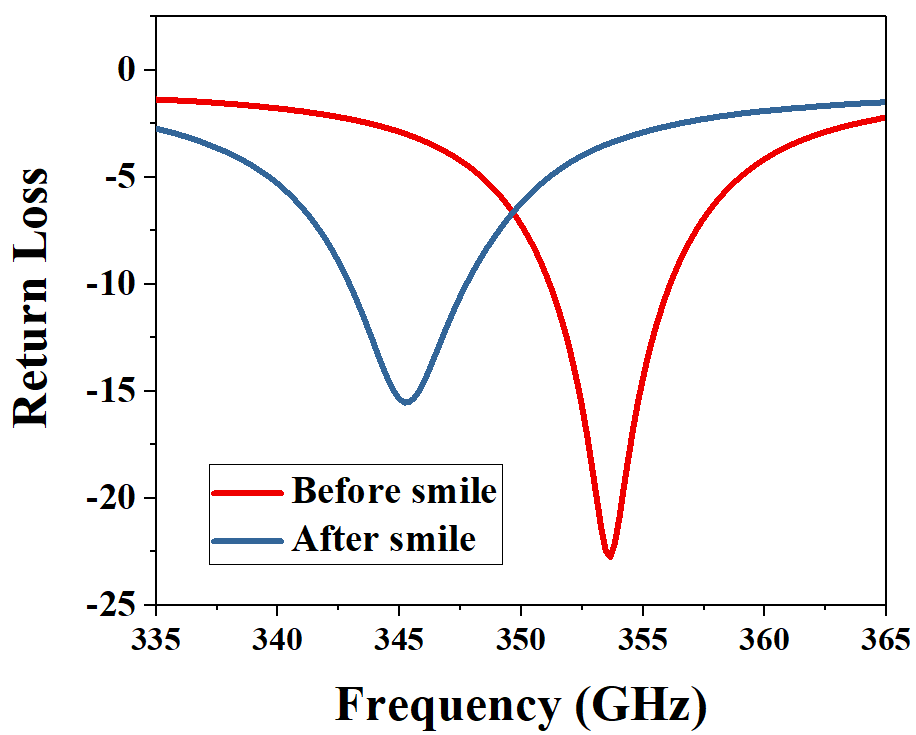


Figure. S8. The variation of resonant frequency before and after smile.


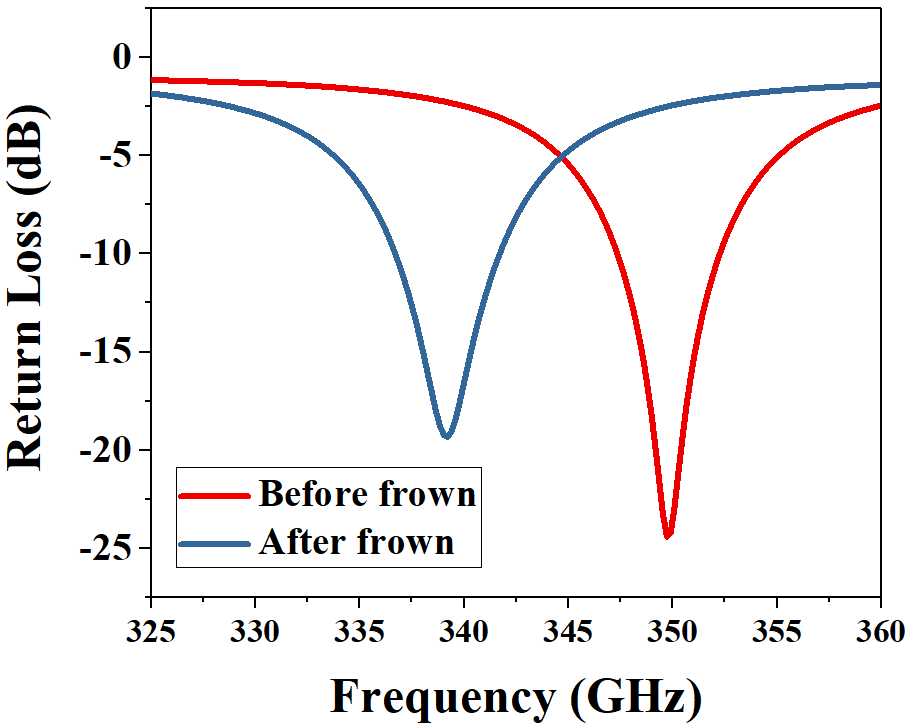


Figure. S9. The variation of resonant frequency before and after frown.
